# Gentry’s Record of the Homœopathic Materia Medica

**Published:** 1895-02

**Authors:** 


GENTRY'S RECORD OF THE HOMŒOPATHIC MATERIA MEDICA. William D. Gentry, M. D., Editor and Publisher, 209 State Street, Room 20, Chicago, Ill. Subscription, $3.00 per year, in advance.

This journal, intended as a companion to the *Concordance Repertory*, is devoted exclusively to Materia Medica.

It is a pamphlet of from 25 to 30 pages, of quarto size, arranged in double column. It contains only materia medica, and the remedies are arranged in alphabetical order. By carefully preserving the numbers as they are published, the subscriber will have at the end of the year a volume of materia medica ready for immediate use. The numbers could be held together by Ballard's Klips, as shown in our advertising pages.

